# Comparison of the Diagnostic Performance of Five Clinical Questionnaires for Chronic Obstructive Pulmonary Disease

**DOI:** 10.1155/2023/2821056

**Published:** 2023-11-24

**Authors:** Alirio R. Bastidas, Eduardo Tuta-Quintero, José S. Arias, Diana Cufiño, Diana Moya, Daniel Martin, Faure Rodríguez, Carolina Aponte-Murcia, Doris M. Pumarejo, Maria A. Bejarano, Geraldine Ospina, Lina M. Morales, Adriana M. Portella, Maria D. Barragán, Daniela A. Álvarez, José M. Hernández

**Affiliations:** ^1^School of Medicine, Universidad de La Sabana, Chía, Colombia; ^2^Clínica Universidad de La Sabana, Chía, Colombia

## Abstract

**Background:**

Chronic obstructive pulmonary disease (COPD) remains one of the most prevalent pathologies in the world and is among the leading causes of mortality and morbidity, partially due to underdiagnosis. The use of clinical questionnaires to identify high-risk individuals to take them to further diagnostic procedures has emerged as a strategy to address this problem.

**Objective:**

To compare the performance of the COULD IT BE COPD, CDQ, COPD-PS, LFQ, and PUMA questionnaires for COPD diagnosis.

**Methods:**

A cross-sectional study was carried out on subjects who underwent spirometry in the third-level center. Data were collected between January 2015 and March 2020. Bivariate analysis was performed between the study variables and the presence of COPD. The area under the receiver operating characteristics curve (AUC-ROC), sensitivity, specificity, positive predictive value (PPV), negative predictive value (NPV), positive likelihood ratio (LR+), and negative likelihood ratio (LR−) for each questionnaire were calculated. The AUC-ROCs were compared with the DeLong test, considering a *p* value <0.05 statistically significant.

**Results:**

681 subjects met the inclusion criteria and were taken to the final analysis. The prevalence of COPD was 27.5% (187/681). The mean age of the subjects was 65.9 years (SD ± 11.79); 46.3% (315/681) were female, and 83.6% (569/681) reported respiratory symptoms. Statistically significant relationship was found for COPD diagnosis with male sex, older age, respiratory symptoms, and exposure to wood smoke (*p* value <0.05). The AUC-ROCs of the questionnaires were between 0.581 and 0.681. The COULD IT BE COPD questionnaire had a lower discriminatory capacity AUC-ROC of 0.581, concerning the other scores (DeLong test, *p* = 0.0002).

**Conclusion:**

The CDQ, COPD-PS, LFQ, PUMA, and COULD IT BE COPD questionnaires have acceptable performance for the diagnosis of COPD together with low sensitivity and specificity. Therefore, its use must be complemented with other diagnostic tests or techniques such as pulmonary function tests.

## 1. Introduction

Chronic obstructive pulmonary disease (COPD) remains one of the most prevalent pathologies in the world, with 212.3 million cases reported in 2019 [1]. Despite the improvement in diagnosis and treatment in the last decades, it continues to be in the top etiologies of morbidity and mortality. In the population above 50 and 75 years old, COPD occupies the 3rd and 4th most frequent cause of disability-adjusted life years (DALYs) and the third most common cause of death [1, 2]. Although statistical information about the morbidity and mortality of COPD in the Latin American population is scarce, the most comprehensive studies show prevalence ranging from 8.9 to 19.4%, with a high rate of underdiagnosis of 87.4% that resembles worldwide literature [3–5].

The universally high rate of COPD underdiagnosis is related to less severe forms of the disease, low educational level, and poor access to pulmonary function tests; these issues seem to be more pronounced in low-resource settings [6]. There is limited information on the health burden of COPD underdiagnosis; nevertheless, the available evidence shows that lung-function decline befalls predominantly in the early stages of the disease [7] and that a late diagnosis impacts the quality of life and clinical outcomes of these individuals [8]. Given the abovementioned issues, multiple case-finding strategies have been developed for the diagnosis of COPD and most of them include the use of clinical questionnaires in the process [9, 10].

One of the most extensively studied questionnaires is the CDQ published in 2006 by Price et al. The researchers developed a score based on eight questions that included risk factors, symptoms, and anthropometric variables. It reported good discriminatory capacity for COPD diagnosis with an AUC-ROC of 0.816 [11]. These findings are not replicated in other populations according to a recent systematic review published in 2016, where the reported AUC-ROC was between 0.65 and 0.71 [12]. More recently, the COPD-PS and LFQ questionnaires were developed based on national surveys conducted in the United States; these investigations found a good discriminatory capacity for COPD diagnosis with a reported AUC-ROC of 0.81 and 0.72 [13, 14]. Both questionnaires were validated in other populations, showing reductions in their performance capacity with an AUC-ROC of 0.748 in the COPD-PS and 0.652 in the LFQ [15, 16].

Available questionnaires include risk factors and symptoms that allow identifying high-risk individuals to take them to further diagnostic procedures. Estimates show that using these scores could translate into a reduction in the number of spirometries necessary to detect cases of COPD, which is relevant when this resource is limited [17]. Among the most used scores in primary care are the COULD IT BE COPD, CDQ, COPD-PS, LFQ, and PUMA [12, 14, 17, 18]. Few of these scores have been compared in other studies showing mixed results [19–21]. None of these studies have included the application of these five questionnaires at the same time in a single population. In addition, the diagnostic performance of the questionnaires changes according to where they are applied, whereby this study aims to compare the diagnostic performance of these five questionnaires in our population.

## 2. Materials and Methods

A cross-sectional study was carried out on subjects who underwent spirometry pre- and post-beta-2-agonist administration in the third-level center Clínica Universidad de La Sabana (Chia, Cundinamarca, Colombia). Data were collected between January 2015 and March 2020.

### 2.1. Selection Criteria

Inclusion criteria included age over 40 years, individuals scheduled to perform spirometry regardless of medical indication, informed consent signature for voluntary participation in the study, native Spanish language speaker, and availability of time to complete the five questionnaires. Subjects whose spirometry did not meet the quality criteria according to the guidelines of the American Thoracic Society (ATS), submitted incomplete questionnaires, and with a limitation for communication or understanding the questionnaire or spirometry technique were excluded [22].

### 2.2. Variables

The data obtained included identification variables, sex, height, weight, race, education level, presence of respiratory symptoms, age at the onset of respiratory symptoms, history of smoking, year package index, exposure to wood smoke, passive smoking, and prior history of COPD. The questionnaires were administered and pre- and post-beta-2-agonist (B2) spirometry was performed, measuring the forced vital capacity (FVC), forced expiratory volume in the first second (FEV1), and the FEV1/FVC ratio, as well as the percentage change of FVC and FEV1. The spirometry was performed with prior calibration of the equipment by the same train and qualified personnel of the pulmonary function laboratory. COPD was defined as the presence of fixed airflow obstruction defined by the American Thoracic Society as the FEV1/FVC ratio <0.7 after bronchodilator administration [22].

### 2.3. Sample Size

Estimation of the sample size for the comparison of areas under the receiver operating characteristics curve (AUC-ROC) was made with the method proposed by Hanley and McNeil [23]. The assumed ratio between no COPD and COPD individuals was 3 : 1, considering the prevalence of COPD in Colombia (26.4%) found by the PUMA study performed in Latin America [24]. The expected AUC-ROC of the questionnaires was taken from the original development studies that reported values of 0.81 and 0.72 [13, 14]. For a confidence interval of 95% and power of 95%, a minimum of 660 individuals were required.

### 2.4. Statistical Analysis

The data were obtained automatically through an electronic collection form, which was automatically entered into an Excel spreadsheet for later verification of their values by the research group for the search for transcription and correction errors. The database was analyzed in the licensed statistical program STATA 14 and SPSS 25. Qualitative variables were reported in frequencies and percentages, and quantitative variables were summarized in the mean and standard deviation. Univariate analysis was performed between the study variables and the presence or absence of COPD using the chi-square test for qualitative variables and Student's *t*-test for quantitative variables. Using the scores obtained from the five questionnaires, AUC-ROC, sensitivity, specificity, positive predictive value (PPV), negative predictive value (NPV), positive likelihood ratio (LR+), and negative likelihood ratio (LR−) for the best cutoff point according to Youden's index were determined. The AUC-ROCs of the different scores were compared with the DeLong test. For the estimates, a *p* value <0.05 was considered significant.

### 2.5. Ethical Considerations

This study was approved by the Research Committee of the Universidad de La Sabana and the Institutional Ethics Committee of the Clínica Universidad de La Sabana, as registered on Act Minute no. 20180928 of September 28, 2022.

## 3. Results

**Figure 1:**
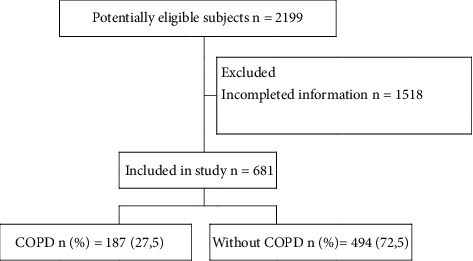
Flowchart showing the selection of subjects and reasons for exclusion from statistical data analysis. COPD: chronic obstructive pulmonary disease.

A total of 2199 potentially eligible patients were admitted during the study period. 681 subjects met inclusion criteria and were taken to the final analysis.  Figure 1 shows the flowchart for the inclusion of the subjects in the study. The prevalence of COPD was 27.5% (187/681).

### 3.1. Population Characteristics

The mean age of the subjects was 65.9 years (SD ± 11.79); 46.3% (315/681) were female, and the mean years of academic training were 8.2 years (SD ± 5.62). 83.6% (569/681) reported respiratory symptoms, and the most frequent one was dyspnea in 64.2% (437/681) of the cases. 44.6% of the subjects (304/681) had a smoking history with a mean index package/year (IPA) of 15.7 (SD ± 26.06). Significant relationship was found for COPD diagnosis with male sex 61% vs. 40.7% (*p* < 0.001), dyspnea 72.2% vs. 61.1% (*p* = 0.007), cough and expectoration 49.2% vs. 39.5% (*p* < 0.001), wheezing 40.6% vs. 28.3% (*p* = 0.002), exposure to wood smoke 69% vs. 54.3% (*p* = 0.001), and prior diagnosis of COPD 50.3% vs. 23.3% (*p* < 0.001). In addition, subjects with COPD were older, 70.8 vs. 64 (*p* < 0.001), and had less years of academic training, 6.8 vs. 8.7 (*p* < 0.001). Further characteristics of the population are summarized in Table 1.

### 3.2. Pulmonary Function

In subjects with COPD, the mean ratio of forced expiratory volume in the first second (FEV1) to forced vital capacity (FVC) was 57.9 (SD ± 10.04) and the predicted FEV1 was 68.8% (SD ± 20.82) pre-B2 and 77.3% (SD ± 20.79) post-B2. Spirometry results of the total population are summarized in Table 2.

### 3.3. Questionnaires Performance for COPD Diagnosis

**Figure 2:**
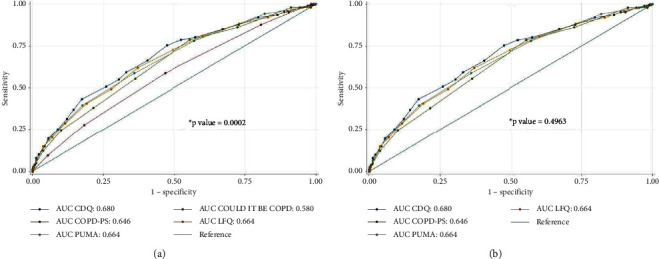
Comparison of the diagnostic performance for COPD among COULD IT BE COPD, CDQ, COPD-PS, LFQ, and PUMA questionnaires. (a) Comparison between the five questionnaires. (b) Comparison between questionnaires excluding COULD IT BE COPD. AUC: area under the receiver operating characteristics curve. ^*∗*^*p* value result of the DeLong test.

The questionnaires with the highest sensitivity corresponded to COPD-PS (78.6%, CI 95%: 75.5–87.5) and the CDQ with (78.6%, CI 95%: 75.5–81.7). The PUMA with a cutoff point of 5 withheld the top specificity (64.2%, CI 95%: 60.6–67.8), but there was a reduction in its sensitivity (58.8%, CI 95%: 55.1–62.5) (Table 3). The AUC-ROC for COPD diagnosis of the questionnaires was between 0.581 and 0.681; the curves are plotted in Figure 2. The comparison of the diagnostic performance between the five questionnaires showed a statistically significant difference with the DeLong test, *p* = 0.0002. A new comparison of the AUC-ROC was performed removing the least accurate questionnaire (COULD IT BE COPD). The analysis showed no difference between the diagnosis performance of the CDQ, COPD-PS, LFQ, and PUMA with DeLong tests, *p* = 0.4963.

## 4. Discussion

In this study, we compared the performance characteristics of five different questionnaires for COPD diagnosis at the same time in a single population. The main finding was that the discriminatory capacity of the CDQ, COPD-PS, LFQ, and PUMA was comparable with an AUC-ROC between 0.646 and 0.681. Comparatively, the COULD IT BE COPD questionnaire had limited discriminatory capacity with respect to the other available scores, with an AUC-ROC of 0.581. Other findings included the determination of a prevalence of 27.5% of COPD in our population and its relationship with older age, male sex, fewer years of academic training, respiratory symptoms, and exposure to wood smoke.

The complexity and costs associated with lung function tests have limited their widespread use, which has led to underdiagnosis and suboptimal management of COPD, especially in primary care practice [6, 25]. To address this issue, in the last two decades, researchers developed clinical questionnaires to identify high-risk individuals to take them to spirometry and demonstrate airflow obstruction distinctive to this disease. The broad spectrum of clinical presentation, severity, and risk factors associated with COPD is a challenge to develop a universally effective screening questionnaire; therefore, available questionnaires are designed from different populations [11, 13, 14, 18, 24].

In 2005, Calverley and collaborators developed one of the first clinical questionnaires for COPD based on the population derived from the Third National Health and Nutrition Examination Survey (NHANES III) [18]. This five-question questionnaire (COULD IT BE COPD) demonstrated good discriminatory capacity for predicting airflow obstruction compatible with COPD with a sensitivity of 85% and NPV of 88%. These results contrast with the findings of our study, where the sensitivity and NPV dropped to 58.8% and 77.4%, and the discriminatory capacity was poor with an AUC-ROC of 0.581. The limited diagnostic performance observed in our study of the COULD IT BE COPD questionnaire could be explained by the noninclusion among the questions of key risk factors identified in our population, such as male sex, stratified age, exposure to wood smoke, and educational level [4].

A prior study that assessed the LFQ questionnaire in our population showed similar performance compared to the original development study with an AUC-ROC of 0.71 [26]. At the moment, only one questionnaire has been developed and validated based on a Latin American population which corresponds to the PUMA score. The original development study in 2016 reported good diagnostic performance with an AUC-ROC of 0.71. Despite the fact that the population on which this questionnaire was developed corresponded to hospitalized subjects, the discriminatory capacity was similar in the external validity studies carried out on primary care and the general population [24, 27]. The available evidence of the performance characteristics of the questionnaires described above shows a clear trend towards a decrease in its discriminative capacities for COPD when evaluated in other populations. The results of our study mostly resemble the findings of the performance characteristics of these questionnaires found in the external validation studies. The difference concerning the original development studies is probably due the variability in the prevalence of disease and distribution of risk factors. These differences originate in the selection of population and inclusion criteria used in each of these investigations; the most relevant is that most external validations were performed on individuals who attended primary care and not in the general population.

Few comparison studies of multiple clinical questionnaires for COPD diagnosis are available in the literature; none of them were performed in Latin America [19–21]. In 2016, Sogbetun and collaborators compared five clinical questionnaires on 383 subjects in primary care in the United States, including COPD-PS and LFQ. [20]. They found a similar discriminatory capacity between the questionnaires with an AUC-ROC of 0.623 for the COPD-PS and 0.655 for the LFQ, which is consistent with our results. The similarity of the discriminative capacities of the questionnaires found in both studies is explained by the resemblance between some of the population features. These include the prevalence of COPD (27.1% vs. 27.5%), a high proportion of subjects with respiratory symptoms (78.2% vs. 83.6%), predominance of older adults, similar lung function in COPD (VEF1/FVC 54% vs. 57.9%), and no COPD (VEF1/FVC 76.2% vs. 75.5%) subjects. The differences in our study should be highlighted, such as a lower proportion of men and a lower prevalence of smoking history.

In 2022, a study comparing multiple questionnaires that included the CDQ, COPD-PS, and LFQ was published by Zhou et al. [21]. The researchers found a comparable diagnostic accuracy among the three questionnaires in a Chinese primary care population with AUC-ROCs between 0.653 and 0.719 and negative predictive values above 85%, which resemble the results of our study. Although the study had a higher prevalence of COPD (36.3%), the distribution of risk factors was similar, with the participants being 45.8% men and 38.8% with smoking history. The available evidence seems to show that the performance of the most comprehensive questionnaires that include and stratify risk factors such as age, the intensity of exposure to smoking, and gender is similar in various populations. Taking into account that the application of the questionnaires evaluated in our study could be interchangeable based on their similar discriminatory capacity for COPD, the intrinsic characteristics of each questionnaire (number of questions, clarity, amount of time to carry it out, etc.) should be evaluated to choose the one with the feasible applicability in the primary care.

Our study has some limitations that must be noted for interpreting its findings. First, it is a single-center study that limits the external validation of its results. In addition, a higher prevalence of COPD compared to current data and a high number of subjects undergoing pulmonary function tests included in our study impact the predictive values and performance found. Nevertheless, the internal validity strengthens by the application of the five questionnaires at the same time in a single population. However, the principal strength of the questionnaires corresponds to their NPV, which would favor their use in populations with a lower prevalence of COPD. It is beyond the scope of this study to propose risk factors for COPD that could improve the discriminatory capacity of the questionnaires, but the noninclusion of risk factors such as exposure to wood smoke and the educational level could have impacted their performance. It is desirable to carry out studies in this direction, as well as measure the cost-effectiveness of screening strategies in our population.

## 5. Conclusion

The CDQ, COPD-PS, LFQ, PUMA, and COULD IT BE COPD questionnaires have acceptable performance for the diagnosis of COPD, together with low sensitivity and specificity. Therefore, its use must be complemented with other diagnostic tests or techniques such as pulmonary function tests.

## Figures and Tables

**Table 1:** General characteristics of the population.

	Total population *n* = 681	COPD *n* = 187	No COPD *n* = 494	*p* value
Age in years, mean (SD)	65.9 (11.8)	70.8 (11.1)	64 (11.5)	<0.001
Males, *n* (%)	315 (46.3)	114 (61)	201 (40.7)	<0.001
Academic training in years, mean (SD)	8.2 (5.6)	6.8 (5.5)	8.7 (5.6)	<0.001
Age at onset of symptoms, mean (SD)	57.9 (16.2)	59.4 (18.1)	57.3 (15.3)	0.180
Dyspnea, *n* (%)	437 (64.2)	135 (72.2)	302 (61.1)	0.007
Cough and expectoration, *n* (%)	287 (42.1)	92 (49.2)	195 (39.5)	<0.001
Wheezing, *n* (%)	216 (31.7)	76 (40.6)	140 (28.3)	0.002
Smoking history, *n* (%)	304 (44.6)	93 (49.7)	211 (42.7)	0.100
IPA, mean (SD)	15.7 (26.1)	19.4 (27.6)	14.2 (25.3)	0.122
Passive smoker, *n* (%)	145 (21.3)	36 (19.3)	109 (22.1)	0.423
Passive smoker years of exposure, mean (SD)	26.2 (17.1)	21.4 (14.5)	27.8 (17.6)	0.055
Exposure to wood smoke, *n* (%)	397 (58.3)	129 (69)	268 (54.3)	0.001
Wood smoke years of exposure, mean (SD)	24.5 (19.6)	26.2 (21.3)	23.7 (18.7)	0.269
Prior diagnosis of COPD, *n* (%)	**209 (30.7)**	**94 (50.3)**	**115 (23.3)**	**<0.001**

COPD: chronic obstructive pulmonary disease, SD: standard deviation, and IPA: index package/year. The *p* value prior diagnosis of COPD, *n* (%) in Table 1 is <0.001. The significance level of the bold values is <0.05.

**Table 2:** General pulmonary function characteristics of study population.

	Total population *n* = 681	COPD *n* = 187	No COPD *n* = 494	*p* value
Weight in kg, mean (SD)	71.3 (13.6)	69.3 (13.8)	72.1 (13.4)	0.014
Height in cm, mean (SD)	159.6 (9.2)	160.3 (9.1)	159.3 (9.3)	0.185

FVC% predicted pre-B2, mean (SD)	95.1 (19.14)	90.6 (19.9)	96.7 (18.6)	<0.001
FVC% predicted post-B2, mean (SD)	97.9 (18.1)	97.5 (19.8)	98 (17.5)	0.736
FEV1% predicted pre-B2, mean (SD)	87.5 (22.6)	68.8 (20.8)	94.5 (18.1)	<0.001
FEV1% predicted post-B2, mean (SD)	93.4 (21.1)	77.3 (20.8)	99.3 (17.9)	<0.001
FEV1/FVC pre-B2%, mean (SD)	70.8 (12.1)	57.9 (10.1)	75.5 (8.8)	<0.001
FEV1/FVC post-B2%, mean (SD)	73.8 (10.6)	59.8 (8.4)	79 (5.3)	<0.001

Z score FVC pre-B2, mean (SD)	−0.2 (1.59)	−0.6 (1.53)	0.1 (1.59)	<0.001
Z score FVC post-B2, mean (SD)	−0.1 (1.39)	−0.1 (1.68)	0.1 (1.27)	0.664
Z score FEV1 pre-B2, mean (SD)	−0.7 (1.67)	−2.0 (1.56)	−0.2 (1.42)	<0.001
Z score FEV1 post-B2, mean (SD)	−0.4 (1.5)	−1.5 (1.42)	0.1 (1.28)	<0.001
Z score FEV1/FVC pre-B2, mean (SD)	−0.5 (1.0)	−1.6 (1.05)	−0.2 (0.62)	<0.001
Z score FEV1/FVC post-B2, mean (SD)	−0.3 (0.92)	−1.4 (0.84)	0.1 (0.49)	<0.001

COPD: chronic obstructive pulmonary disease, kg: kilograms, SD: standard deviation, cm: centimeters, COPD: chronic obstructive pulmonary disease, B2: beta-2-agonist, FVC: forced vital capacity, and FEV1: forced expiratory volume in the first second.

**Table 3:** Performance of COPD diagnostic questionnaires in study population.

Questionnaire cutoff points	Se (CI 95%)	Sp (CI 95%)	VPP (CI 95%)	VPN (CI 95%)	LR+ (CI 95%)	LR−· (CI 95%)	AUC (CI 95%)	*p*value
LFQ ≤18	60.1 (56.4–63.8)	19.3 (16.3–22.2)	66.3 (62.7–69.8)	15.5 (12.7–18.2)	1.34 (0.98–1.83)	0.48 (0.35–0.66)	0.66 (0.67–0.71)	<0.001
CDQ ≥16	78.6 (75.5–81.7)	47.4 (43.6–51.1)	42.8 (39.0–46.5)	81.6 (78.7–84.S)	1.49 (1.33–1.67)	0.45 (0.40–0.50)	0.68 (0.61−0.73)	<0.001
PUMA ≥S	58.8 (55.1–62.5)	64.2 (60.67.8)	38.3 (34.7–42.0)	80.5 (77.5–83.4)	1.64 (1.38–1.94)	0.64 (0.54–0.75)	0.67 (0.62–0.71)	<0.001
COULD IT BE COPD ≥3	58.8 (55.1–62.5)	53.2 (49.5–57)	32.3 (28.7–35.8)	77.4 (74.2–80.5)	1.26 (1.08–1.46)	0.77 (0.66–0.90)	0.58 (0.61–0.63)	0.001
COPD-PS ≥4	78.6 (75.5–87.5)	44.3 (33.5–55.2)	34.8 (24.5–45.2)	84.6 (76.7–92.4)	1.41 (1.26–1.57)	0.48 (0.43–0.53)	0.65 (0.66–0.69)	<0.001

Se, sensitivity; Sp, specificity; PPV, positive predictive value; NPV, negative predictive value; LR+, positive likelihood ratio; LR−, negative likelihood ratio; AUC, area under the receiver operating characteristic curve.

## Data Availability

The data used to support the findings of this study are available from the corresponding author upon request.

## Abbreviations

COPD: Chronic obstructive pulmonary disease
AUC-ROC: Area under the receiver operating characteristics curve
PPV: Positive predictive value
PPN: Negative predictive value
LR+: Negative likelihood ratio
LR−: Negative likelihood ratio
ATS: American Thoracic Society
B2: Beta-2-agonist
FVC: Forced vital capacity
FEV1: Forced expiratory volume in the first second.
